# Deep Learning‐Assisted Fingerprint‐Inspired Flexible Pressure Sensor for Tension Monitoring in Carbon Fiber Production

**DOI:** 10.1002/advs.202513680

**Published:** 2025-09-30

**Authors:** Xiaohua Wu, Xiangbao Huang, Yuxuan Liang, Longsheng Lu, Shu Yang, Jiayue Liao, Hanxian Chen, Feilong Liu, Yilin Zhong, Qinghua Liang, Yingxi Xie

**Affiliations:** ^1^ School of Mechanical & Automotive Engineering South China University of Technology 381 Wushan Road, Tianhe District Guangzhou Guangdong 510640 China

**Keywords:** anomaly classification, carbon fiber production, fiber tension monitoring, flexible pressure sensor, laser manufacturing

## Abstract

Carbon fiber has become a key emerging material in fields such as aerospace, wind power generation, and new energy vehicles. However, the current mass production of carbon fiber is limited by the challenges in controlling tension stability across wide‐width carbon fiber tow arrays, which restricts the performance and quality stability of the final product. Inspired by ancient textile workers who used their fingers to feel the tension of fiber bundles, a flexible pressure sensor is fabricated via laser etching to mimic the structure of fingerprints for monitoring the tension of wide‐width carbon fiber bundle arrays. This sensor demonstrates high sensitivity (18.08 kPa^−1^) and a broad pressure range of 320 kPa, with a maximum measurable pressure of 550 kPa. By installing the sensor array on the surface of a tension roller, multi‐fiber tension detection is achieved with a sensitivity of 5.55 N^−1^. Furthermore, an end‐to‐end tension anomaly classification convolutional neural network is developed and achieved a high classification accuracy of 96%. This sensor offers a practical solution for real‐time, intelligent tension monitoring in carbon fiber production, which helps address key technical challenges in large‐scale, high‐consistency manufacturing and provides a new application for advancing sensing technology in flexible electronics and smart manufacturing.

## Graphical abstract

A fingerprint‐inspired piezoresistive sensor is developed for real‐time tension monitoring of wide‐width carbon fiber bundles. Fabricated by laser engraving, it shows high sensitivity, broad range, and durability under high‐temperature conditions. Integrated with a CNN achieving 96% anomaly classification accuracy, the system provides a practical solution for stable, intelligent, and large‐scale carbon fiber manufacturing.

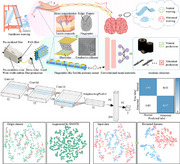

## 1 Introduction

Sensors can accurately convert physical signals into electrical outputs, serving as a key intelligent component in the industrialization of high‐end products,^[^ ^1^, ^2^, ^3^ ^]^ such as carbon fiber.^[^ ^4^ ^]^ During the pre‐oxidation stage of carbon fiber production, both physical and chemical shrinkage occur. Applying sufficient tension to the polyacrylonitrile fibers at this stage effectively suppresses shrinkage, promotes molecular alignment, and enhances the mechanical performance of the final product.^[^ ^5^, ^6^ ^]^ Therefore, tension control plays a crucial role in ensuring the stability and quality of carbon fiber manufacturing.

Currently, fiber bundle tension in wide‐width carbon fiber production lines is mainly regulated by the creel and drive rollers (Figure S1, Supporting Information). In practical operations, variations in fiber bundle tension often arise due to complex chemical reactions during pre‐oxidation.^[^ ^7^, ^8^ ^]^ Moreover, the dense arrangement of multiple fiber bundles in wide‐width production lines poses significant challenges to individual tension monitoring. Due to the lack of effective real‐time monitoring tools, it is difficult to precisely regulate the tension of each fiber bundle, which negatively affects the consistency and performance of the final carbon fiber products. Therefore, developing a sensor that can accurately monitor the tension of fiber bundles during wide‐width carbon fiber production is of significant engineering value, which would enhance the stability and consistent performance of carbon fiber products.

Fiber tension sensors are generally divided into two categories: non‐contact^[^ ^9^, ^10^, ^11^ ^]^ and contact types.^[^ ^12^, ^13^, ^14^ ^]^ Non‐contact sensors often suffer from high cost, limited integration capability, slow response speed, and vulnerability to environmental disturbances. In contrast, contact tension sensors are more widely adopted and have become the primary devices used for monitoring fiber bundle tension in practical carbon fiber production lines.^[^ ^15^, ^16^, ^17^, ^18^ ^]^ Their working principle is usually based on the three‐pulley system, where the fiber bundle is sequentially routed over auxiliary and sensing pulleys.^[^ ^19^, ^20^ ^]^ The applied tension exerts a radial force on the sensing pulley, causing displacement or strain in the attached sensor. However, contact tension sensors are often bulky and difficult to integrate, making it challenging to monitor the tension of multiple fiber bundles arranged in the same plane during the pre‐oxidation stage.

As early as the era of manual weaving, textile workers monitored bundle tension using their fingertips, where fingerprint patterns helped concentrate stress onto tactile receptors. Inspired by this, a fingerprint‐inspired flexible pressure sensor is proposed for real‐time tension measurement of multiple carbon fiber bundles. The sensor consists of an interdigital electrode (IDE) on a flexible printed circuit board (FPCB), a polyamide (PA) film adhesive layer, a fingerprint‐like sensing layer, and a polyimide (PI) film encapsulation layer. The fingerprint‐like sensing layer is fabricated by laser engraving conductive silicone, followed by multi‐step hot pressing to complete the sensor assembly. Flexible pressure sensors are generally manufactured using soft, conductive materials such as elastomers,^[^ ^21^ ^]^ conductive polymers,^[^ ^22^ ^]^ or composites,^[^ ^23^ ^]^ which provide high flexibility and conformability, enabling thin, lightweight, and easily integrable devices. Compared with other fabrication methods such as micro‐molding,^[^ ^24^ ^]^ soft lithography,^[^ ^25^ ^]^ or printing‐based techniques,^[^ ^26^ ^]^ laser engraving allows precise patterning of complex microscale structures, ensures high reproducibility, and facilitates rapid prototyping of sensors with customizable geometries. Additionally, hot pressing ensures uniform bonding of sensor layers and stable mechanical properties, which are crucial for high‐sensitivity pressure detection. The proposed sensor exhibits a high sensitivity that 18.08 kPa^−1^ in the 8–320 kPa range and 5.46 kPa^−1^ in the 320–550 kPa range. The sensor was successfully applied in a simulated carbon fiber production system with expandable bundles, demonstrating a tension sensitivity of 5.55 N^−1^ within the 2–18 N. Owing to the perpendicular fingerprint‐like micro ridge, the sensor exhibits insensitivity to tangential forces. Under small tangential disturbances, the signal variation remains minimal, enabling accurate measurement of carbon fiber tension without interference from friction. To further verify its practical application potential, we developed an end‐to‐end tension anomaly classification model, achieving a classification accuracy of 96%. We believe this fingerprint‐inspired flexible pressure sensor can enable independent, real‐time monitoring of fiber bundle tension of wide‐width carbon fiber production. This work lays the foundation for intelligent tension control in carbon fiber manufacturing, providing crucial support for ensuring product performance and production consistency.

## 2 Results

### 2.1 Design and Working Mechanism of Fingerprint‐Inspired Flexible Pressure Sensor

**Figure 1:**
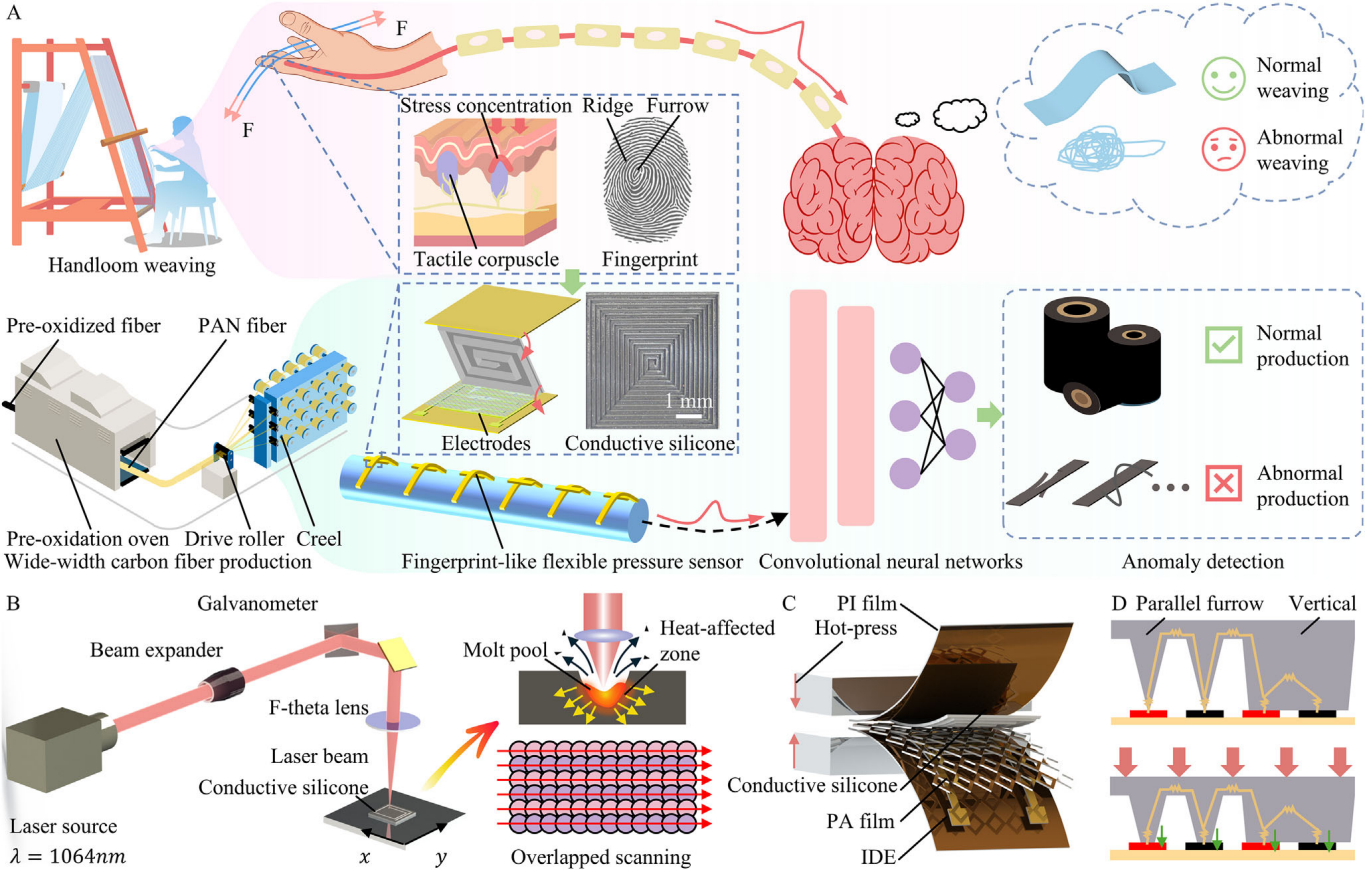
Schematic illustration of the fingerprint‐inspired flexible pressure sensor. A) The comparison of tension sensing and anomaly classification methods between traditional handloom weaving and modern carbon fiber production processes. B) Schematic of laser engraving on the conductive silicone to create a fingerprint‐like sensing layer. C) Illustration of the encapsulation process of the sensor via hot pressing, consisting of a PI film, conductive silicone sensing layer, adhesive PA film, and IDE on FPCB. D) Working principle of the fingerprint‐inspired flexible pressure sensor.

As shown in **Figure** **1**A, early textile workers assessed silk bundle tension by touching the yarn with their fingertips while operating the handloom. The ridges and furrows in the fingerprint concentrated the applied stress onto tactile receptors, which then triggered action potentials transmitted to the brain.^[^ ^27^, ^28^ ^]^ Based on this feedback, the brain could identify whether the tension was normal or abnormal, helping ensure a smooth and consistent textile process.

Inspired by this biological mechanism, we propose a fingerprint‐inspired flexible pressure sensor to address the current lack of effective real‐time tension monitoring methods in large‐width carbon fiber production. The sensor consists primarily of a pressure‐sensing layer and an IDE. The surface of the sensing layer features horizontal and vertical fingerprint‐like furrows, which modulate the contact area with the interpolation electrodes under applied pressure. In human fingerprints, the spiral ridges have evolved specific geometric parameters, which are closely matched to the distribution and responsiveness of tactile receptors at the fingertip. Guided by these biological insights, we optimized the spacing and depth of the sensor's micro ridges to mimic this fine mechanical modulation, thereby improving sensitivity and maintaining stable contact under varying pressures. These electrodes mimic the role of human tactile receptors and nerves by converting pressure changes into variations in electrical signals. Human fingerprints are spiral rather than parallel linear patterns, which helps prevent the ridges from toppling under omnidirectional tangential forces and avoids false perception of normal pressure in unloaded regions. Similarly, in carbon fiber tension detection, unavoidable friction exists. By designing perpendicular micro ridges, the structure resists tilting under tangential forces, thereby greatly reducing the influence of friction (Figure S2, Supporting Information).

By integrating this sensor onto the surface of the tension roller, we can obtain real‐time measurements of both the variation and magnitude of fiber bundle tension during operation. To overcome the limitations of traditional hand‐crafted feature extraction, such as poor generalization, low sensitivity to waveform anomalies, and time‐consuming design, we further introduce an end‐to‐end tension anomaly classification model based on convolutional neural networks (CNN). This model directly processes raw sensor signals without manual feature engineering and outputs a judgment of whether the tension state in the carbon fiber production process is normal or abnormal.

As shown in Figure 1B, the sensing layer is fabricated by laser engraving conductive silicone rubber. The strong photothermal effect of the laser creates micro‐melting pools at the focal points and heat‐affected zone in the surrounding. By overlapping these melt zones, a fingerprint‐like furrow is formed on the sensing layer surface. As illustrated in Figure 1C, an IDE, a PI film, the fingerprint‐like sensing layer, an adhesive PA film, and an IDE are assembled into a flexible sensor using a hot‐pressing process (Figure S3, Supporting Information). In the absence of pressure, only the peaks of the ridge structures contact the electrode, allowing minimal current to flow. Upon applying pressure, the sensing layer compresses, increasing the contact area and generating additional conductive paths between the ridges and the electrodes. This leads to a significant reduction in contact resistance, as shown in Figure 1D. The result is a pronounced increase in response current, demonstrating an electrical signal that corresponds well with the applied pressure.

### 2.2 Simulation and Analysis of Different Microstructures

**Figure 2:**
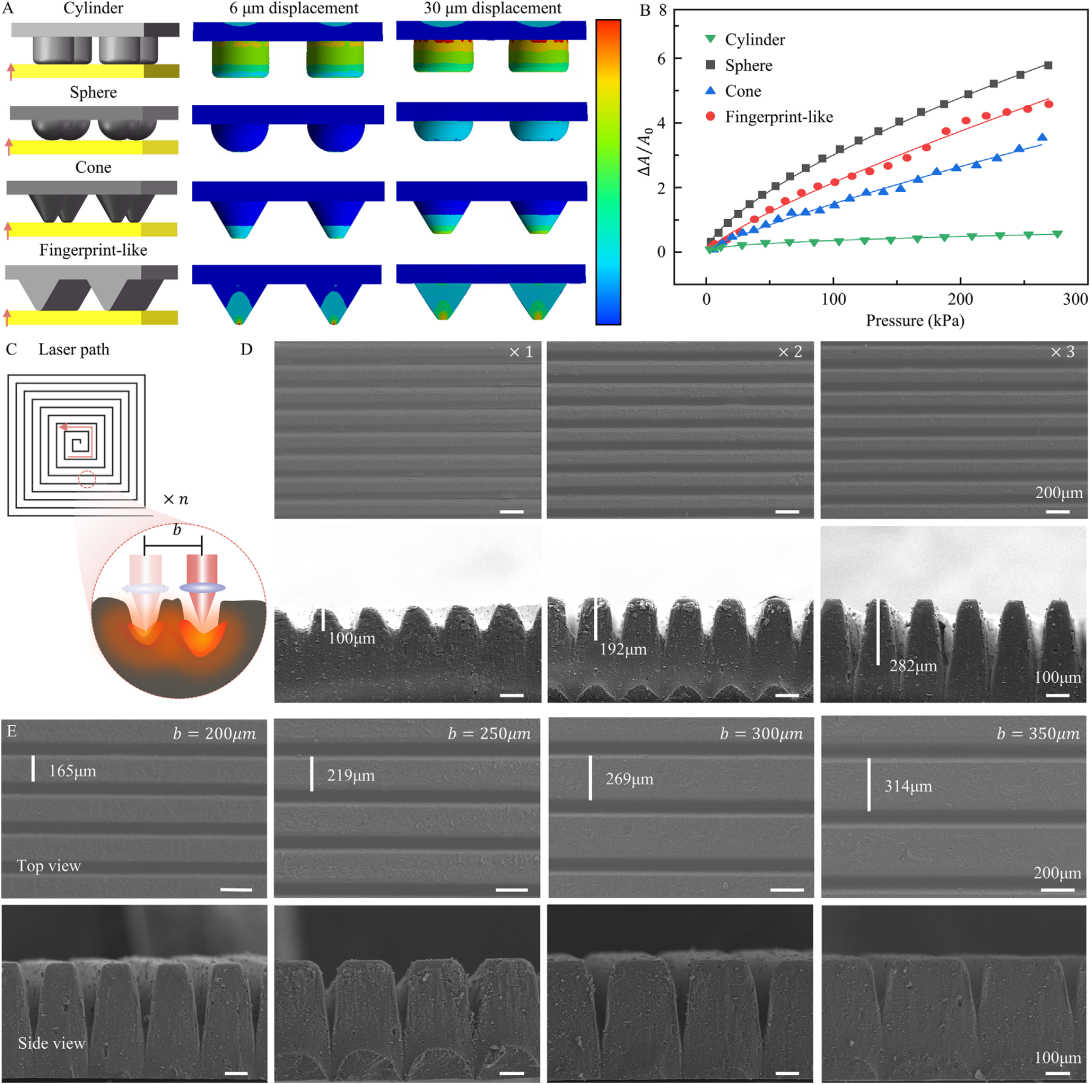
Finite element simulation and laser processing analysis of different microstructures. A) Finite element models of typical cylindrical, hemispherical, conical, and the proposed fingerprint‐like structures were established to analyze the stress fields under varying levels of applied pressure. B) Relationship between contact area and applied pressure for the four types of microstructures. C) Schematic diagram of laser processing a fingerprint‐like groove. Effects of the number of laser passes D) and spiral pitches E) on the formation of fingerprint‐like structures.

To evaluate the performance advantages of the fingerprint‐like structure in flexible pressure sensors, we conducted finite element mechanical simulations on commonly used microstructures. As shown in **Figure** **2**A, models of four different sensing layer structures were developed, including cylindrical, hemispherical, conical, and fingerprint‐like structures. The schematic of dividing the finite element simulation mesh is shown in Figure S4 (Supporting Information). The stress concentration and distribution in each sensing layer under applied pressure were analyzed to compare their mechanical response characteristics.

Simulation results showing the stress distribution at electrode displacements of 6 and 30 µm are presented in Figure 2A. The stress of the cylindrical structure was mainly concentrated at the junction between the microstructure and the substrate. In contrast, the hemispherical, conical, and fingerprint‐like structures exhibited stress concentration primarily at the contact interface with the electrode layer. Under the same electrode displacement, the cylindrical structure showed minimal change in contact area, whereas the contact surfaces of the other three structures undergo more significant deformation.

We further extracted the contact force and contact area between the sensing and electrode layers. By converting the contact force to an external load, we analyzed how the contact area changes with increasing load. Figure 2B shows the simulated relationship between the contact area change rate and external load for each structure, using the contact area at 3 kPa as the initial contact area. Among the four structures, the hemispherical exhibits the highest rate of change in contact area, followed by the fingerprint‐like, conical, and cylindrical structures in descending order. The relationship between surface area changes and external load for each structure was fitted using a power function, and the corresponding mathematical expressions were obtained:

(1) $$\Delta A/A_{0}=0.050p^{0.423}\left(\mathrm{Cylindrical}\right)$$

(2) $$\Delta A/A_{0}=0.137p^{0.669}\left(\mathrm{Hemispherical}\right)$$

(3) $$\Delta A/A_{0}=0.033p^{0.827}\left(\mathrm{Conical}\right)$$

(4) $$\Delta A/A_{0}=0.053p^{0.800}\left(\mathrm{Fingerprint}-\mathrm{like}\right)$$

here, *p* denotes the applied pressure load on the sensor, and Δ*A*/*A*_0_ represents the rate of change in contact area. The power function fitting results show that the exponents for the cylindrical, hemispherical, conical, and fingerprint‐like structures were 0.423, 0.669, 0.827, and 0.800, respectively. These results indicated that the conical and fingerprint‐like structures exhibit a more linear relationship between contact area change rate and applied load. Furthermore, the fingerprint‐like structure showed a higher contact area change rate compared to the conical structure, suggesting improved sensitivity under pressure. Since the relationship between contact area variation and applied pressure directly influences the linearity and sensitivity of piezoresistive sensors, the fingerprint‐like sensor demonstrates excellent performance across a wide pressure range.

### 2.3 Laser Engraving of Sensing Layers

Using fiber laser to engrave conductive silicone, the detailed laser parameters are shown in Table S1 (Supporting Information). Due to the Gaussian distribution of laser energy, the highest laser energy is concentrated in the central spot region, with the energy gradually decreasing toward both sides. Uneven energy distribution can lead to incomplete material removal in recessed areas. One effective way to achieve a more uniform laser energy distribution across the processing area is to adjust the spacing between parallel laser paths, thereby controlling the overlap rate of the laser spots (Figure S5A, Supporting Information). The overlap rate of the laser spots along the direction parallel to the scan path is defined as:

(5) $$K=\frac{2r-w}{2r}\times 100\%$$

here, *r* represents the radius of the laser beam spot, and *w* is the distance between the two parallel paths. To study the effect of varying laser spot overlap rates on the ablation of graphite nickel‐plated silicone rubber, experiments were carried out using a laser power of 10 W and a scanning speed of 200 mm s^−1^. The laser processing was performed with overlap rates of 70%, 60%, 40%, and 20%, with six parallel lines scanned for each condition.

The processing results are shown in Figure S5B (Supporting Information). As the overlap rate increases, the processed furrows become narrower and deeper. When the overlap rate drops below 40%, periodically raised stripes appear within the grooves, indicating incomplete material removal. In contrast, higher overlap rates produce deeper but narrower grooves due to the localized concentration of laser energy. To balance processing efficiency with the formation of well‐defined microstructures, a laser overlap rate of 60%, corresponding to a 20 µm spacing between parallel paths, was chosen for the direct laser writing of the fingerprint‐like sensing layer in the subsequent sensor fabrication.

The laser beam moves in a spot scanning mode along a helical path during engraving. The shape of the fingerprint‐like structure significantly influences its pressure resistance and consequently affects the variation in contact area between the furrows and the interpolation electrode. The ridge shape is determined by its width and height (Figure 2C). To create ridges with different shapes, we controlled the ridges width by adjusting the spacing between the helices in the laser processing pattern, and varied the number of laser passes to change the depth of the furrows, thus forming ridges of different heights.

Accordingly, different helix group spacings and numbers of laser passes were designed to prepare the sensing layers with fingerprint‐like structures. As shown in Figure 2D, SEM images display the micro‐ridged structures fabricated with varying numbers of laser passes. The laser‐processed parallel helixes form V‐shaped furrows, leaving unprocessed areas between them that are wider at the base and narrower at the top, creating the raised micro‐ridges. With an increasing number of laser passes, the ridge height increases while the tip becomes sharper. The ridge heights measured ≈100, 192, and 282 µm for one, two, and three passes, respectively. The growth in ridge height slowed with additional passes, likely due to laser energy absorption by the protrusions and energy scattering caused by deviations of the sample surface from the laser's focal point, which reduced material removal beneath the ridges. At the same number of passes, the ridges showed consistent height and width, indicating uniformity of the prepared sensing layer across the entire area.

Figure 2E shows micro‐ridged structures produced by laser direct writing with different spiral pitches. Varying the spiral pitches changes the ridge morphology: smaller spiral pitches result in longer, narrower ridges, while larger spacings produce ridges with wider bases and tops. The ridges formed at larger spiral pitches also tend to have greater heights and widths when in contact with the interpolation electrode, confirming the uniformity of the sensing layer's fingerprint‐like structure throughout. These fingerprint‐like structures exhibit a high initial contact area with the interpolation electrode. Notably, at a helix spiral pitch of 400 µm, the ridges showed relatively lower height compared to those formed at other spacings, possibly because wider ridges absorb more laser energy during engraving.

### 2.4 Characterization of Fingerprint‐Inspired Flexible Pressure Sensor

**Figure 3:**
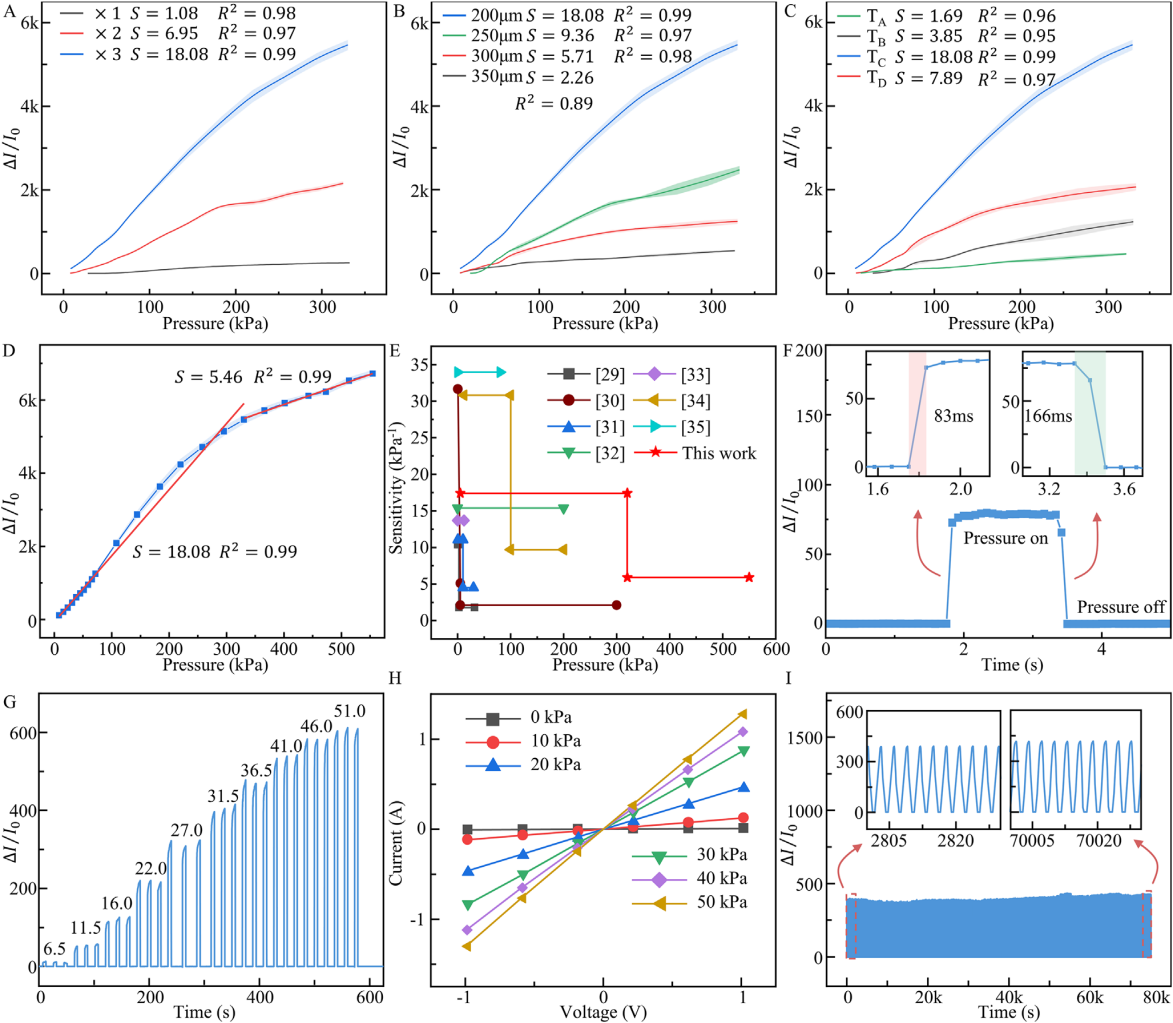
Performance of the fingerprint‐inspired flexible pressure sensor. A) Relationship between the current change rate and pressure for sensors fabricated with varying numbers of laser passes. B) different pattern spiral pitches. C) and interpolation electrode widths. D) Current change rate versus pressure of the sensor fabricated under the optimized process conditions. The dynamic sensing characteristic tests were repeated three times to reduce random errors, and the error bars represent mean ± standard error (SE). E) Performance comparison between the developed fingerprint‐inspired flexible pressure sensor and other reported resistive‐type flexible pressure sensors. F) Response and recovery times of the sensor. G) Current responses under different applied pressures. H) Current responses of the sensor at various operating voltages. I) Repeatability test results over 25 000 pressure cycles.

After fabrication of the fingerprint‐inspired flexible pressure sensor, its performance was systematically characterized. As shown in **Figure** **3**A, within the pressure range of 8–320 kPa, the sensor's sensitivity increases with the number of laser passes. The sensing layer subjected to three laser passes achieved the highest sensitivity of 18.08 kPa^−1^, whereas the sensor processed only once exhibited the lowest sensitivity of 1.08 kPa^−1^. At a constant ridge width, a greater ridge height leads to larger deformation under pressure. Among the samples, the sensor processed three times had the tallest micro‐ridges, which deformed more easily, resulting in a larger change in contact area between the sensing layer and interpolation electrode, consequently, higher sensitivity. In contrast, the sensors processed only once or twice had lower ridge heights, smaller deformations under load, and less variation in current response. Although increasing ridge height improves sensor sensitivity, excessive laser processing can damage the conductive silicone rubber substrate. Therefore, the sensing layer processed three times was selected for optimal performance and reliability.

As shown in Figure 3B, when the width of the micro‐ridges increases, the rate of change in output current under the same pressure decreases. Sensors fabricated with spiral pitches of 200, 250, 300, and 350 µm exhibited sensitivities of 18.08, 9.36, 5.71, and 2.26 kPa^−1^, respectively. The corresponding linear correlation coefficients (R^2^) were 0.99, 0.95, 0.98, and 0.89, indicating good linearity. Wider spiral pitch results in broader ridge tops, increasing the initial contact area with the electrode layer. This leads to a higher initial output current and a relatively smaller current change under pressure. In contrast, the 200 µm‐spacing design produces narrower ridge tips, yielding a lower initial current and a larger relative increase in output current when pressure is applied. Additionally, a smaller spiral pitch results in more densely packed ridges within the same sensing area. This increases the number of parallel resistive pathways in contact with the electrode layer, further enhancing the current response under loading. However, denser and thinner ridge structures may have reduced resilience, limiting recovery after deformation. Taking these factors into account, a square helix pattern with 200 µm spiral pitch was selected as the optimal configuration for fabricating the sensing layer using fiber laser processing.

The interpolation electrode refers to the structural configuration of the electrode layer, which influences the contact area with the sensing layer and determines the conductive paths, thereby affecting the performance of the piezoresistive sensor. To study the effect of electrode design, sensors were fabricated using four different electrode structures, labeled A, B, C, and D, differing in single electrode width and number of electrode pairs (Figure S6 and Table S2, Supporting Information). In type A, B, and C, the width of individual electrodes increased successively, while structure D maintained the same electrode width as structure C but had only half the number of electrode pairs. These electrode widths correspond to a gradual increase in fill factor (30%, 40%, 60%, 77%) and W/g ratio (0.43, 0.67, 1.50, 3.35).

As shown in Figure 3C, the sensors using structures A, B, C, and D exhibited sensitivities of 1.69, 3.85, 18.08, and 7.89 kPa^−1^, respectively, with corresponding linear R^2^ of 0.96, 0.95, 0.99, and 0.97. These results indicate that when the electrode filling ratio is too low (type A), the effective contact between the electrodes and the micro‐ridged sensing layer is insufficient, thereby limiting sensitivity. As the filling ratio increases with larger width gap ratio (types B and C), the contact interaction improves, leading to enhanced sensitivity. However, in type D, despite having a larger filling ratio than type C, the reduced number of electrode pairs increases the effective spacing between adjacent electrodes, which decreases the number of parallel conductive pathways and lowers sensitivity. Therefore, type C, with a balanced electrode width (1.5 mm electrode width with 7 pairs), was determined to be the optimal configuration, as it provides sufficient coverage and appropriate inter‐electrode spacing while maintaining multiple conduction paths.

To further evaluate the performance of the optimized sensor, the pressure range was expanded. As shown in Figure 3D, the sensor exhibited a sensitivity of 18.08 kPa^−1^ in the 8–320 kPa range with a linear fit (R^2^ = 0.99), and a sensitivity of 5.46 kPa^−1^ in the 320–550 kPa range (R^2^ = 0.99). Compared to previously reported high sensitivity piezoresistive sensors, which often show a limited sensing range or rapidly decreasing sensitivity at high pressures (Figure 3E; Table S3, Supporting Information^[^ ^29^, ^30^, ^31^, ^32^, ^33^, ^34^, ^35^ ^]^), the sensor developed in this study maintains excellent linearity and high sensitivity across a wide pressure range. This is attributed to the optimized contact area formed between the fingerprint‐like sensing layer and the interpolation electrode. Overall, the sensor demonstrates reliable performance over a broad pressure range, with high sensitivity and linear response. It is well‐suited for detecting small pressure changes and a wide pressure range, such as those occurring between carbon fiber bundles and tension rollers, enabling accurate monitoring of fiber tension in real‐time during production.

A consistent piezoresistive sensor is characterized by highly uniform electrical output signals from different sensors under the same pressure load, which improves calibration efficiency and interchangeability. However, due to fabrication processes and manual assembly, sensors prepared under identical parameters may still exhibit certain performance variations. To evaluate the consistency of the fabricated sensors, four sensor units produced under the same parameters were tested for their current variation rates under different pressures, as shown in Figure S7 (Supporting Information).

The coefficient of variation (CV) of sensitivity was introduced as an indicator of consistency, where a smaller CV value reflects higher consistency among sensors. The CV of sensitivity is calculated as follows:

(6) $$CV=\left(\frac{\sigma }{\mu }\right)\times 100\%$$

where σ is the standard deviation of sensor sensitivity and μ is the mean sensitivity. Based on the sensitivity values, the calculated CV within the pressure range of 0–320 kPa is 3.5%, indicating that the sensors fabricated under identical parameters exhibit high consistency.

The sensor's response and recovery times are shown in Figure 3F. When a pressure of 12 kPa was rapidly applied and released, the sensor exhibited a sharp increase in output current within 83 ms and a decrease within 166 ms, indicating a fast response and recovery. As shown in Figure 3G, the sensor was subjected to multiple loading cycles at different pressures ranging from 6.5 to 51 kPa. The output waveforms remained stable under all pressure levels, and similar current change rates were observed under repeated loading of the same pressure. This suggests that the contact between the sensing layer and the electrode layer remained stable, and the sensor exhibited consistent resistive response behavior. To evaluate performance under varying electrical conditions, the sensor was tested at pressures of 10, 20, 30, 40, and 50 kPa across different operating voltages. The developed sensor can detect small pressures. As shown in Figure S8 (Supporting Information), the sensor accurately responds to a pressure of 250 Pa applied on its surface and returns to the original current afterward.

As shown in Figure 3H, the output current increased linearly with voltage under each pressure condition, confirming that the sensor maintained stable resistance and reliable performance across a range of voltages. Durability testing was conducted using a tensile testing machine, applying a 32 kPa pressure load in repeated loading‐unloading cycles at a rate of one cycle every 3 s. As shown in Figure 3I, the sensor maintained a stable electrical response over 25 000 cycles, demonstrating excellent operational longevity. To verify the stable response of the sensor under conditions similar to those in the carbon fiber production line, the sensor was placed on a heating stage at 80 °C and subjected to repeated pressure tests using a tensile testing machine. The results are shown in Figure S9 (Supporting Information). The sensor exhibited consistent responses (Δ*I*/*I*_0_ = 81.03 ± 0.56). Additionally, the influence of tangential forces on the sensor's performance was evaluated (Figure S10, Supporting Information). Segmented linear fitting of the current change rate versus pressure curves, with and without tangential force, showed high correlation (R^2^ >0.99) in the 0–300 kPa range. Under a tangential force of 0.1 N, the mean deviation in variation rate was 4.01%, and under 0.5 N it was 5.80%. The fingerprint‐like micro‐ridges are arranged in a square spiral pattern, where adjacent perpendicular ridges prevent the ridges from toppling under tangential forces. Additionally, the PA film provides stable bonding between the sensing layer and the electrode layer, preventing relative displacement in the tangential direction. As a result, tangential forces have only a minor effect on the sensor's electrical output, and the designed fingerprint‐like piezoresistive sensor can meet the requirements of carbon fiber bundle tension monitoring applications.

### 2.5 Construction of Fiber Bundle Tension Monitoring System

**Figure 4:**
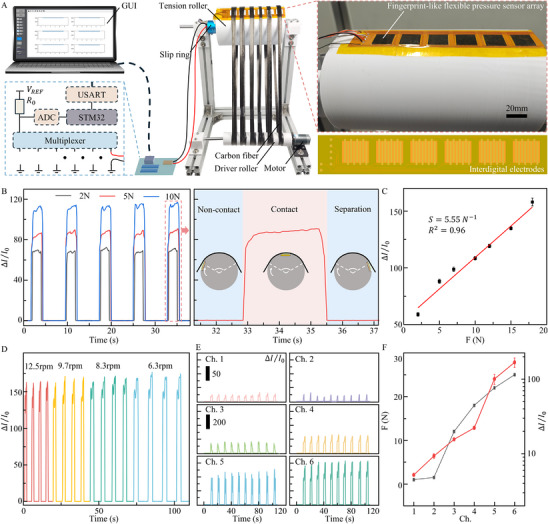
Construction and performance of the tension monitoring system. A) Schematic of the tension monitoring system components. B) Sensor current responses under varying tension levels. C) Correlation between sensor current changes and applied bundle tension. The tests were repeated three times, and the error bars represent mean ± SE. D) Sensor current variations at different rotational speed. E) Comparison of current responses from six sensor channels under different tensions. F) Quantitative relationship between sensor current variation and applied tension. The responses were tested by six channels, and the error bars represent mean ± SE.

As shown in **Figure** **4**A, this study implements individual tension monitoring of multiple fiber bundles by measuring the contact pressure between the fiber bundles and the tension roller using a piezoresistive sensor array integrated on the roller surface. The physical diagram of the developed sensor and its array is shown in Figure S11 (Supporting Information). The monitoring system consisted of a driver roller, a tension roller, a piezoresistive sensor array, a conductive slip ring, a data acquisition hardware circuit, and a graphical user interface (GUI) on a computer. The data acquisition hardware converted resistance changes from the piezoresistive sensors into analog signals via an analog‐to‐digital converter (ADC). In this configuration, a 9 kΩ reference resistor was used in the sampling circuit, as its value is comparable to the typical resistance range of the sensors, thereby maximizing measurement sensitivity while keeping the output within the ADC input range. The output digital signal from the data acquisition unit corresponds to the sensor's resistance, and the relationship between them is given by:

(7) $$Value_{ADC}=4096\frac{R_{S}}{R_{S}+9000}$$

The value of the resistance to be measured is denoted as *R_S_*, and the analog signal obtained from the ADC is denoted as *Value_ADC_*. A comparison between theoretical and measured values is shown in Figure S12 (Supporting Information). The choice of a 9 kΩ reference resistor enables the sampling circuit to achieve high sensitivity and accuracy in measuring the typical resistance range of the sensors (0–100 kΩ). A standard tension was applied to the carbon fiber by hanging calibrated weights on the opposite side of the tension roller (Figure S13, Supporting Information). Tension levels of 2, 5, and 10 N were applied, and the corresponding sensor responses are shown in Figure 4B. As the fiber bundle rotated along with the tension roller shaft, it contacted the integrated fingerprint‐like sensor. During the contact phase, the sensor's output remained stable, indicating that the pressure exerted by the fiber bundle on the sensor was consistent throughout the contact. Once the sensor was no longer in contact with the fiber bundle, the pressure load was removed, and the sensor's output returned to its initial current. Under the same applied tension, the sensor shows similar and repeatable response curves, confirming that the system maintains stable performance under identical conditions.

The developed tension monitoring system was further tested across a range of fiber bundle tensions. The system's average output current change while the fingerprint‐like sensor was in contact with the fiber bundle was used as the response value. The relationship between sensor response and applied tension within the 2–18 N range is shown in Figure 4C. The system demonstrated a sensitivity of 5.55 N^−1^ and a linearity of 0.96, confirming its suitability for practical fiber bundle tension monitoring applications.

To assess its performance under dynamic conditions, the system was tested at various fiber bundle speeds. As shown in Figure 4D, the output waveforms vary slightly with speed, but the response values remain stable. This indicated that the system can reliably monitor fiber tension at different operational speeds. Further, six fiber bundles were subjected to tensions of 1, 1.5, 12, 18, 22, and 25 N, respectively, corresponding to sensing channels 1–6. As shown in Figure 4F, the sensor responses increase progressively with tension, clearly distinguishing the differences between bundles. This proves that the system can effectively detect tension inconsistencies across multiple fiber bundles in real‐time. Such capability provides valuable guidance for adjusting individual bundle tensions, thereby improving tension uniformity and enhancing the overall consistency and quality of carbon fiber production.

### 2.6 Tension Anomaly Classification Task in Carbon Fiber Simulation Production Process

**Figure 5:**
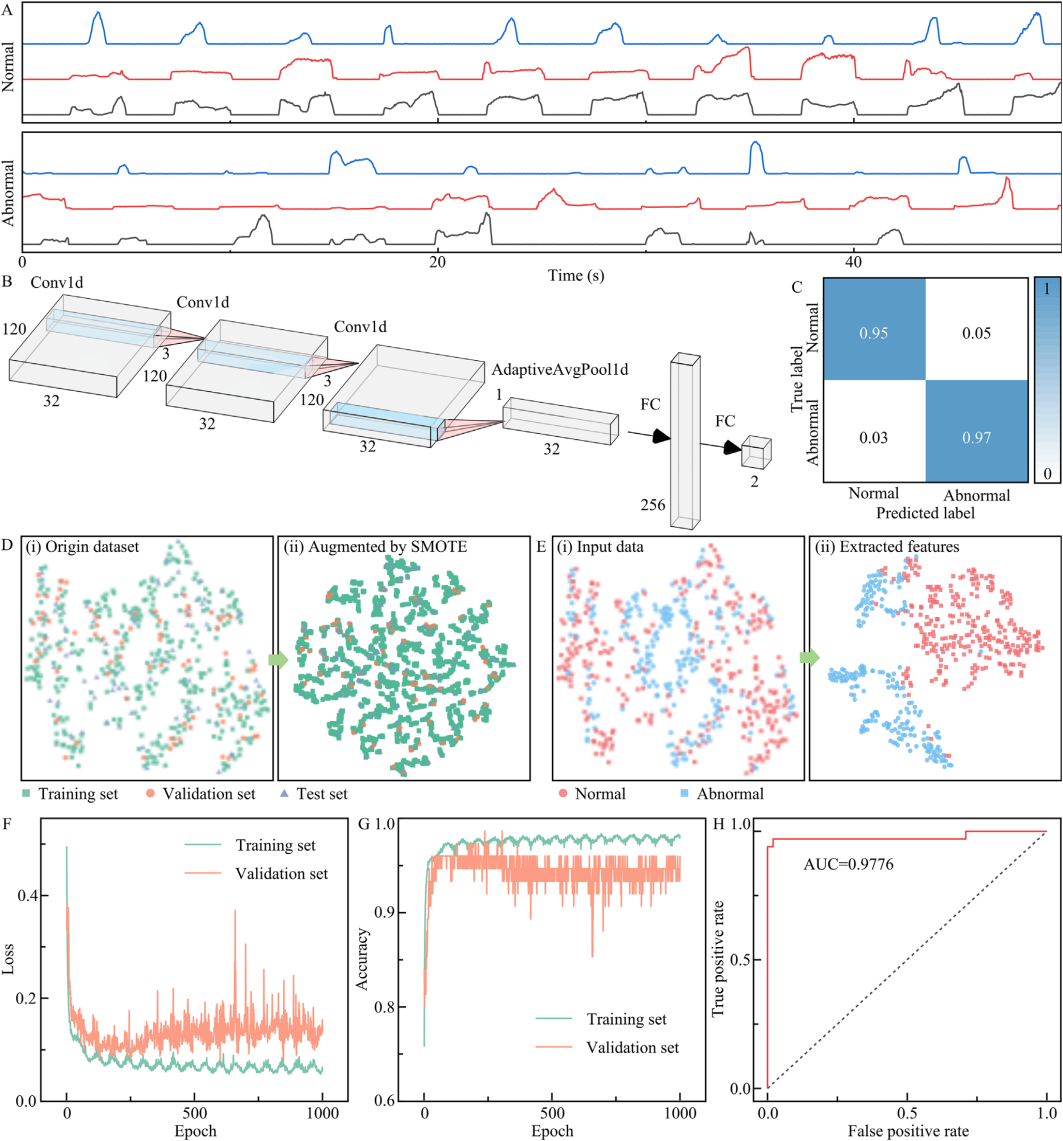
Application in the tension anomaly classification task. A) Sensor output current waveforms under normal and abnormal operating conditions. B) CNN network architecture block diagram. C) Confusion matrix of the model's predictions on the test dataset. D) t‐SNE visualization of data distribution before and after SMOTE augmentation. E) t‐SNE visualization of feature distributions before and after feature extraction by the model. F) Loss curve during the training process of the model. G) Accuracy curve during model training. H) ROC curve of the trained model evaluated on the test dataset.

The above results confirm that the developed fingerprint‐inspired flexible pressure sensor can accurately detect fiber bundle tension. Uneven tension and other abnormal operational events can be effectively identified based on the tension signal. Traditional methods for detecting abnormal signal typically involved identifying tension peaks using wave search or wavelet transform techniques, followed by manually designing algorithms to screen out abnormal signals through statistical analysis. However, these approaches are often complex and require re‐tuning of feature extractors and statistical models when application scenarios or sensor installation positions change. To address this, we propose an end‐to‐end deep learning model for detecting abnormal tension in fiber bundles. As illustrated in **Figure** **5**A, normal and abnormal tension signals are fed into a CNN model. The detailed CNN model and architectural components are shown in Figure 5B and Table S4 (Supporting Information). The model successfully classifies the signals, achieving a clear distinction between normal and abnormal signals (Figure 5C). Details on dataset construction and model training are provided in the experimental section.

The original dataset contains 498 samples, divided into training, validation, and test sets using an 8:1:1 stratified sampling strategy. To enhance model robustness, the training set (348 samples) was expanded to 10 000 samples using the synthetic minority over‐sampling technique (SMOTE) data augmentation technique. Figure 5D compares the t‐distributed stochastic neighbor embedding (t‐SNE) visualizations of the original and augmented datasets. The augmented training set maintains a similar distribution to the original validation and test sets, supporting its representativeness in building model decision boundary. As shown in Figure 5E, although the original data's t‐SNE distribution exhibits significant overlap between normal and abnormal samples, the model's extracted features show clear separation, allowing for reliable classification through the fully connected layers. Figure S14 (Supporting Information) details the t‐SNE distribution of the original data and extracted features under different datasets, further demonstrating the stable classification ability of the trained model.

Figure 5F,G depicts the training loss and accuracy trends. The model began to overfit after ≈250 epochs, as indicated by increasing validation loss. To mitigate this, we applied early stopping and a cosine annealing learning rate schedule to escape local minima and converge toward an optimal solution. During training, the loss and accuracy initially changed rapidly and later exhibited periodic fluctuations due to the cyclic variation of the learning rate, which helps the model escape local optima. This resulted in periodic fluctuations in loss and accuracy every 30 epochs. After training, the model reaches a classification accuracy of 96%, with evaluation metrics summarized in **Table** **1**. The model's overall classification performance is further validated by the receiver operating characteristic (ROC) curve in Figure 5H. An area under the curve (AUC) of 0.9776 confirms excellent classification capability, with a high true positive rate and low false positive rate across different threshold values. To ensure that the trained model did not overfit and produce inflated accuracy, 5 fold cross‐validation was performed. For model training, the training folds in each cross‐validation split were augmented using SMOTE to reach 10 000 instances. Inference was then conducted on the same test set for the five trained models, and detailed performance is shown in Table S5 (Supporting Information). Each model's accuracy was slightly lower than that obtained using the full training set. This reduction is because, although SMOTE expanded the data to 10 000 instances, the amount of real data was reduced by 8.9% compared to the previous training, resulting in a slight decrease in performance. These results demonstrate the strong robustness and generalizability of the proposed tension anomaly classification model.

**Table 1:** Detailed performance of the trained anomaly detection model in the test set.

	Precision	Recall	F1‐score	Support
Normal	0.9756	0.9524	0.9639	42
Abnormal	0.9412	0.9697	0.9552	33
Macro avg	0.9584	0.9610	0.9595	75
Weighted avg	0.9605	0.9600	0.9601	75
Accuracy			0.9600	75
AUC Score			0.9776	

In real‐world applications, more types of damage may occur, such as chemical fractures, incomplete gradient structures, or variations in surface roughness. These forms of damage pose greater challenges for model recognition, as they may require longer time windows to capture or identify complex signal features and complete damage categories. In such cases, more powerful and complex temporal models such as recurrent neural networks (RNNs) and transformer‐based models are often needed to analyze long‐sequence data.

As shown in Figure S15 (Supporting Information), we tested several models, including transformer, long short‐term memory (LSTM), gated recurrent unit, and hybrid CNN‐RNN, on the current dataset. The results indicate that these models achieved performance close to or matching the performance of using a CNN model. This outcome is due to their higher complexity compared with the CNN, while the current sequence length of only 120 data points is too short to fully exploit the advantages of these complex models. However, such methods hold promise for future applications in identifying more complex real‐world anomalies. In addition, a promising approach is to train more comprehensive models on abnormal data that are easier to collect and then transfer part or all of these models to categories where data are more difficult to obtain, thereby improving recognition rates for rare abnormal cases.

## 3 Conclusion

The tension of fiber bundles during the continuous production of carbon fiber directly affects product quality and batch consistency. In wide‐width carbon fiber production lines, the dense arrangement of fiber bundles presents a significant challenge for real‐time tension monitoring. To address this issue, this study proposes a fiber bundle tension monitoring method based on a piezoresistive sensor. Drawing inspiration from the structure of human fingerprints, we designed a piezoresistive sensor featuring a micro‐ridged fingerprint‐like architecture. The spacing and width of human fingerprint ridges have been optimized through biological evolution, playing a critical role in tactile sensitivity and force discrimination. These key biological characteristics were emulated and systematically optimized in the sensor's micro‐ridge design, resulting in enhanced sensitivity and more uniform stress distribution. The sensor was fabricated using laser engraving, tailored to meet the demands of tension monitoring in the carbon fiber production process. The sensor exhibits high sensitivity (18.08 kPa^−1^), a wide working range (8–550 kPa), fast response time (83/166 ms), long cycling life (>25 000 cycles), and a low detection limit (250 Pa). The sensor can operate stably at the high temperature of 80 °C on the pre‐oxidation furnace rollers.

Based on this sensor, a fiber bundle tension monitoring system was developed to enable real‐time tension detection during continuous production. This system achieves a sensitivity of 5.55 N^−1^ within a detection range of 0–18 N. Furthermore, an end‐to‐end anomaly classification CNN was constructed, which accurately identifies abnormal tension signals with an accuracy of 96%. The proposed approach demonstrates strong feasibility and application potential in real production environments. The proposed method demonstrates strong feasibility and application potential in practical production environments.

## 4 Experimental Section

### Sensor Preparation Based on Laser Engraving

A graphite‐nickel‐plated conductive silicone rubber (Qihong Material Co., Ltd.) sheet with a thickness of 0.5 mm was cut into 25 × 25 mm^2^ squares using laser processing. The cut sheets were sequentially cleaned in anhydrous ethanol and deionized water using ultrasonic treatment for 10 min to remove surface contaminants, followed by drying in an oven at 60 °C. A square spiral pattern measuring 20 × 20 mm^2^ with a line width of 200 µm was designed and processed using a fiber laser. The patterning process involved three passes at a scanning speed of 200 mm s^−1^ and a laser power of 10 W. After laser engraving, the laser was used to trim the edges, yielding sensing layer samples with a final size of 20 × 20 mm^2^. Following laser processing, the samples underwent ultrasonic cleaning in anhydrous ethanol for 20 min. Subsequently, a spray gun was used to remove any residual particles on the microstructure surface, and the samples were dried in a 60 °C oven. A grid‐patterned PA film (Figure S3, Supporting Information), chosen for its thermoplastic nature and strong adhesion capability, together with the prepared sensing layer, was bonded to the FPCB electrodes via hot pressing at 160 °C. Finally, the entire sensor assembly was encapsulated using a PI film with adhesive backing, leveraging its high thermal stability, mechanical robustness, and electrical insulation properties to serve as the protective layer.

### Finite Element Simulation Setup

As shown in Figure 2A, three common micro‐patterned structures, including cylindrical, hemispherical, and conical, along with a proposed fingerprint‐like structure, were modeled as sensing layer geometries. All microstructures were designed with identical base widths and heights. In the model, the lower layer serves as the rigid electrode, while the upper layer represents the deformable sensing layer incorporating the microstructures. A representative segment of the sensing layer was modeled to reduce computational load, assuming uniform structure and deformation. Additionally, sharp corners on the cylindrical, conical, and fingerprint‐like structures were rounded to minimize stress concentration artifacts that can cause excessive computation time or lead to simulation non‐convergence. Material parameters for the sensing layer were based on graphite‐nickel‐coated silicone rubber, with a density of 1100 kg m^−^^3^, an elastic modulus of 10 MPa, and a Poisson's ratio of 0.45. The electrode layer, composed of metal, was treated as a rigid body due to its significantly higher stiffness compared to the sensing layer. A frictionless contact interaction was defined between the microstructure sensing surface and the rigid electrode. The microstructure surface was designated as the contact surface, while the electrode surface served as the target surface. Both the sensing layer and electrode contact surface were meshed with an element size of 10 µm using automatic mesh generation. Since the electrode is defined as rigid, only its contact surface requires meshing. Taking the fingerprint‐like structure as an example, the resulting finite element mesh is illustrated in Figure S4 (Supporting Information). A multi‐step simulation procedure was employed, with automatic time stepping enabled. The initial time step was set to 10, with a minimum of 1 and a maximum of 1000. External loading was simulated by applying vertical displacement from the electrode to the sensing layer, with each computational step applying a displacement increment of 0.002 µm.

### Test for Flexible Pressure Sensor

A standard pressure was applied to the sensor using a tensile testing machine (Wise Co., ZQ‐990B), and the corresponding current output was measured with a digital source meter (Keithley 2450). The sensitivity of the sensor was calculated using the following equation:

(8) $$S=\frac{\left(I_{1}-I_{0}\right)/I_{0}}{p}$$

where I_1_ is the current measured after applying pressure, I_0_ is the initial current, and *p* is the applied pressure. To investigate the effect of tangential force on the sensor's response, a controlled tangential force was simulated during tension measurements. This was achieved by attaching a layer of PI film to the surface of the sensor to introduce varying levels of tangential friction force.

### Tension Monitoring Simulator Construction

As shown in Figure 4A, the constructed tension detection simulation system consists of three main components: a mechanical system, a hardware system, and a software system. The mechanical system includes a driver roll, a tension roll, a carbon fiber bundle, a sensor array, and a conductive slip ring. The sensor array is designed with expandable channels to accommodate different numbers of fiber bundles, simulating various real‐world scenarios. As illustrated in Figure S10 (Supporting Information), standard weights could be suspended from the fiber bundles to apply precise tension, enabling accurate calibration and testing of the developed sensors under different tension conditions. A bonded carbon fiber ring is used to simulate the continuous operation typically encountered in actual production environments (Figure 4A). The hardware system consisted of an STM32 microcontroller and a multiplexer chip (CD4067), which enabled asynchronous connection of multiple sensor channels to a shared signal acquisition circuit. A 9 kΩ reference resistor was used in the sampling circuit, as its value is comparable to the typical resistance range of the sensors (0–100 kΩ), thereby ensuring both sensitivity and accuracy in measurement. Analog voltage signals are captured via an ADC, allowing calculation of the current through each sensor. These multi‐channel sensor responses are then transmitted via USART to a PC‐based GUI for real‐time display and data storage.

### Anomaly Classification Dataset Collection and Model Training

A simulation platform was developed to measure fiber bundle tension and to evaluate the sensor's response under cyclic operating conditions. To artificially induce abnormal scenarios, such as snagging, tangling, and slipping, we adopted a simplified setup using a three‐bundle configuration, which allowed for easier manipulation of controlled fault events. During experiments, sensor output currents were recorded separately under normal and abnormal conditions. The collected signals were segmented into non‐overlapping time windows of 4 s to build a complete dataset. This dataset was then randomly divided into training, validation, and test sets at a ratio of 8:1:1. To enhance the robustness of the model, the training set was expanded using the SMOTE data augmentation method. The architecture and parameters of the proposed model are detailed in Figure 5B and Table S4 (Supporting Information).

Model training was constructed on an Apple Mac Mini M4. The model was trained with a learning rate of 10^−3^ for 1000 epochs, using a batch size of 128 and a fixed random seed of 0. The cross‐entropy loss function was used as the loss function, and the Adam optimizer with a weight decay of 10^−3^ was employed to update the parameters. A cosine annealing learning rate schedule was employed with a period of 30 epochs, during which the learning rate decays smoothly from the initial setting to 0 at the end of each cycle. Validation accuracy was monitored at each epoch, and an early stopping strategy was used to prevent overfitting and ensure the selection of the model weights with the best generalization performance. To ensure that the model did not overfit during training, 5 fold cross‐validation was performed. Here, the training and validation sets were combined and then randomly split into 5 folds. SMOTE was applied to the resulting training folds to generate synthetic samples, expanding the dataset to 10 000 instances. The test set used was the same as the above for consistent comparison.

### Statistical Analysis

All quantitative data are presented as the mean ± SE of three experimental measurements to ensure statistical reliability and minimize variability. The sensing characteristics were analyzed using linear fitting to calculate sensitivity, providing a systematic description of the dynamic response. In the experimental study, each dynamic sensing characteristic was tested three times to reduce measurement errors and improve repeatability. Statistical analysis was performed using Origin (OriginLab, USA) and Excel (Microsoft, USA).

## Conflict of Interest

The authors declare no conflict of interest.

## Supporting information



Supporting Information

Supplemental Movie 1

## Data Availability

All data are available in the main text or the supplementary materials.
