# Supplementary material for: Deep Learning‐Assisted Fingerprint‐Inspired Flexible Pressure Sensor for Tension Monitoring in Carbon Fiber Production
Source: Adv Sci (Weinh). 2025 Sep 30;12(47):e13680. doi: 10.1002/advs.202513680 (PMC12713018; doi:10.1002/advs.202513680)
Supplement: Supplementary file 1 — Supporting Information [file ADVS-12-e13680-s001.docx]

**Deep Learning-Assisted Fingerprint-Inspired Flexible Pressure Sensor for Tension Monitoring in Carbon Fiber Production**

Xiaohua Wu^1^, Xiangbao Huang^1^, Yuxuan Liang^1^, Longsheng Lu^1^, Shu Yang^1^, Jiayue Liao^1^, Hanxian Chen^1^, Feilong Liu^1^, Yilin Zhong^1^, Qinghua Liang^1^, Yingxi Xie^1*^

^1^School of Mechanical & Automotive Engineering, South China University of Technology, Guangzhou 510641, China.

^*^Corresponding author. Email: xieyingxi@scut.edu.cn.


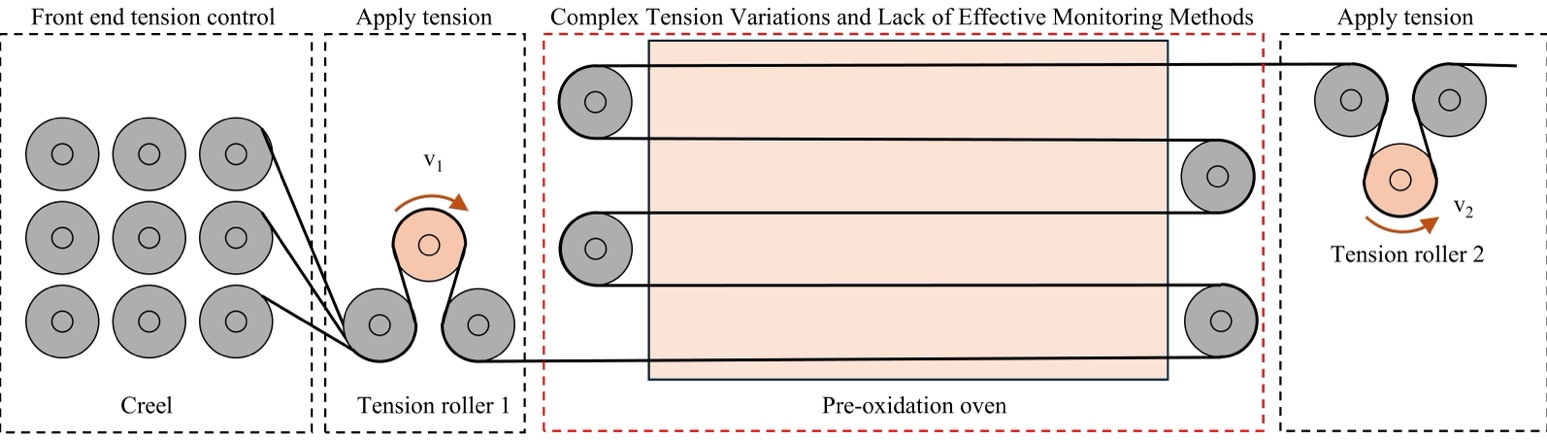
Fig. S1. Wide range of tension control requirements for practical carbon fiber production.


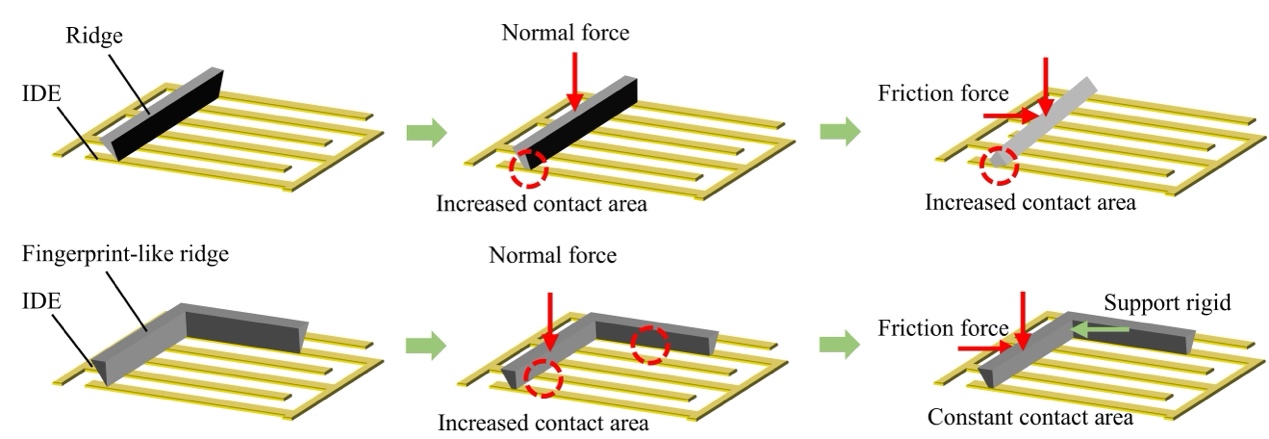


Fig. S2. The perpendicular fingerprint-inspired micro-ridge structure can effectively reduce the coupling effect of friction during carbon fiber tension detection.


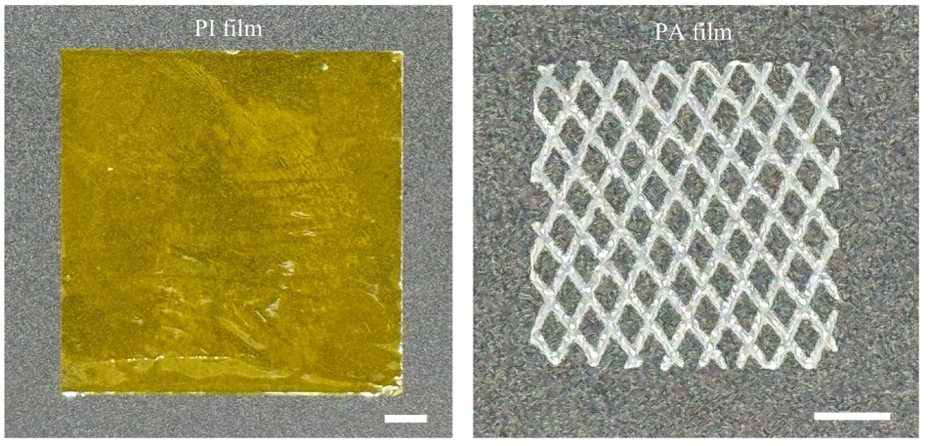


Fig. S3. Photographs of the protective layer (PI film) and adhesive layer (PA film). The scale bar represents 5 mm.


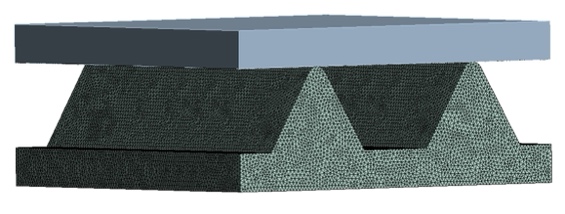


Fig. S4. Simulation model meshing of fingerprint-like structures.


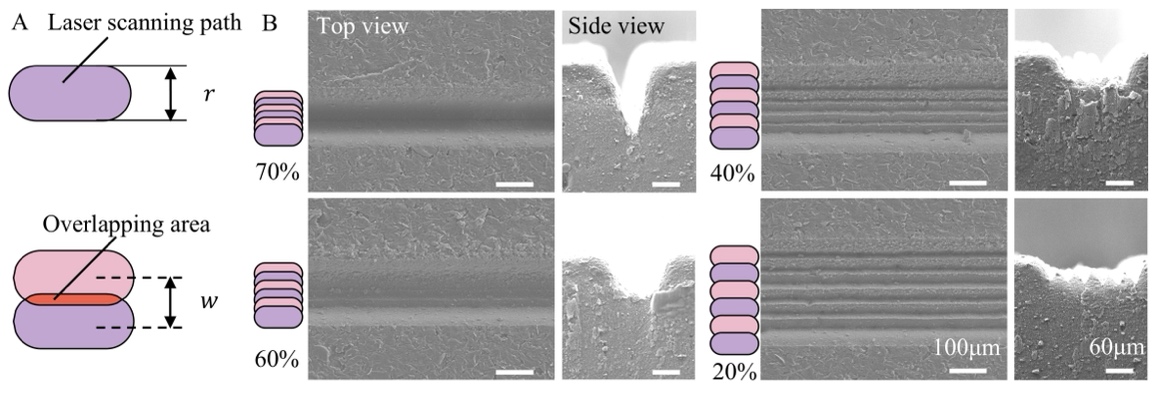


Fig. S5. Discussion on processing with different laser overlap ratios. (A) Schematic diagram of laser overlaps processing. (B) Influence of different laser overlap ratios on the quality of fingerprint ridge fabrication.


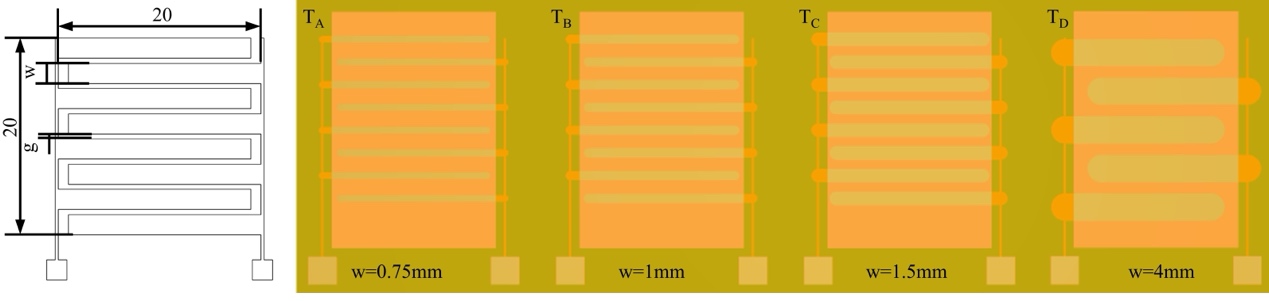


Fig. S6. Schematic diagrams of four different finger insertion electrodes.


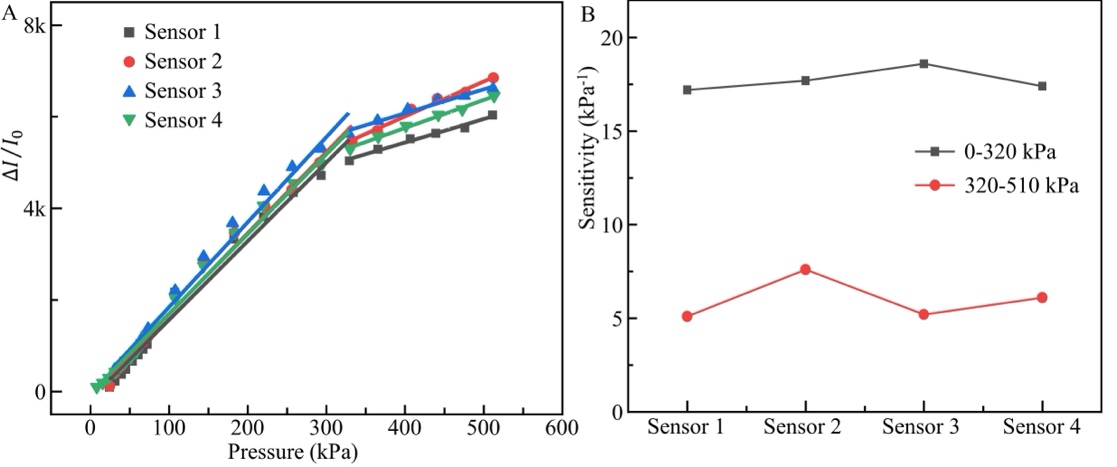


Fig. S7. Consistency of sensors. (A) Current rate of change-pressure curve. (B) Sensor sensitivity.


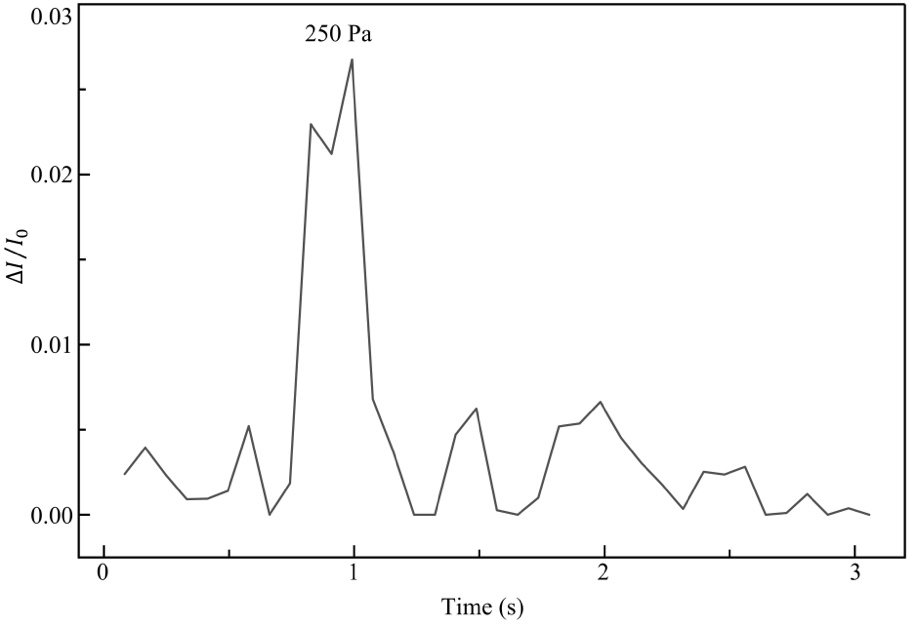


Fig. S8. The minimum detection limit of the developed sensor.


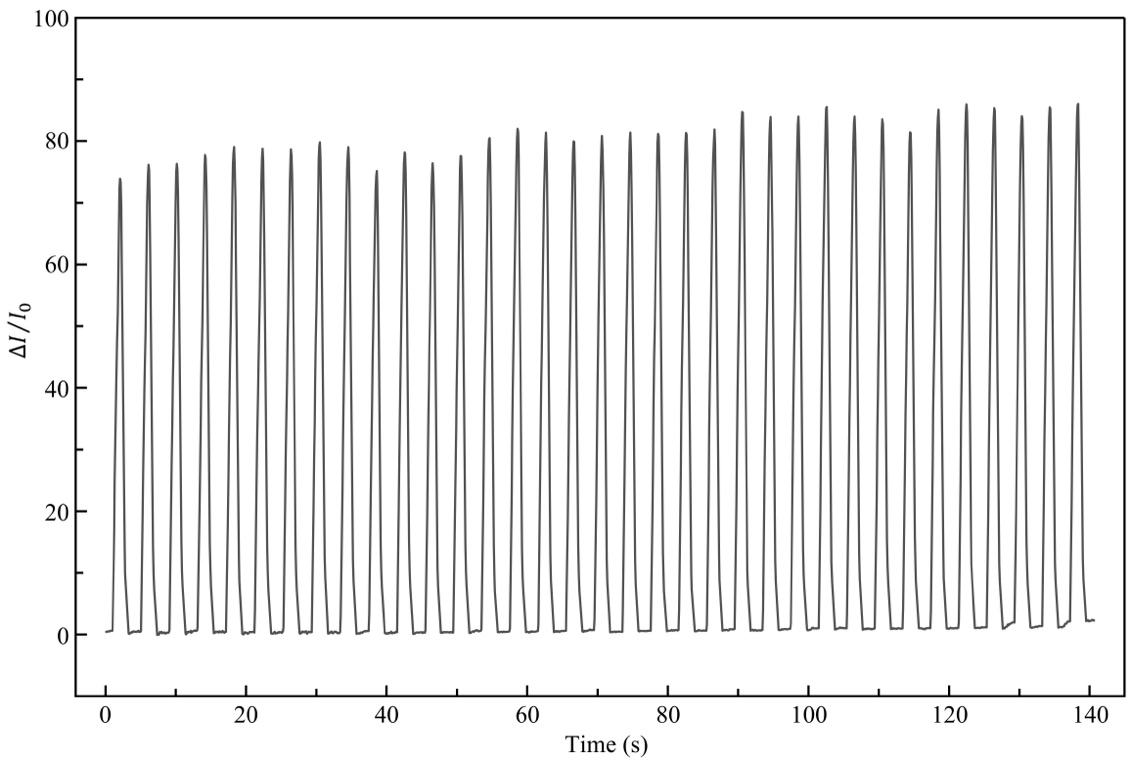


Fig. S9. Sensor response under normal operation at 80 °C.


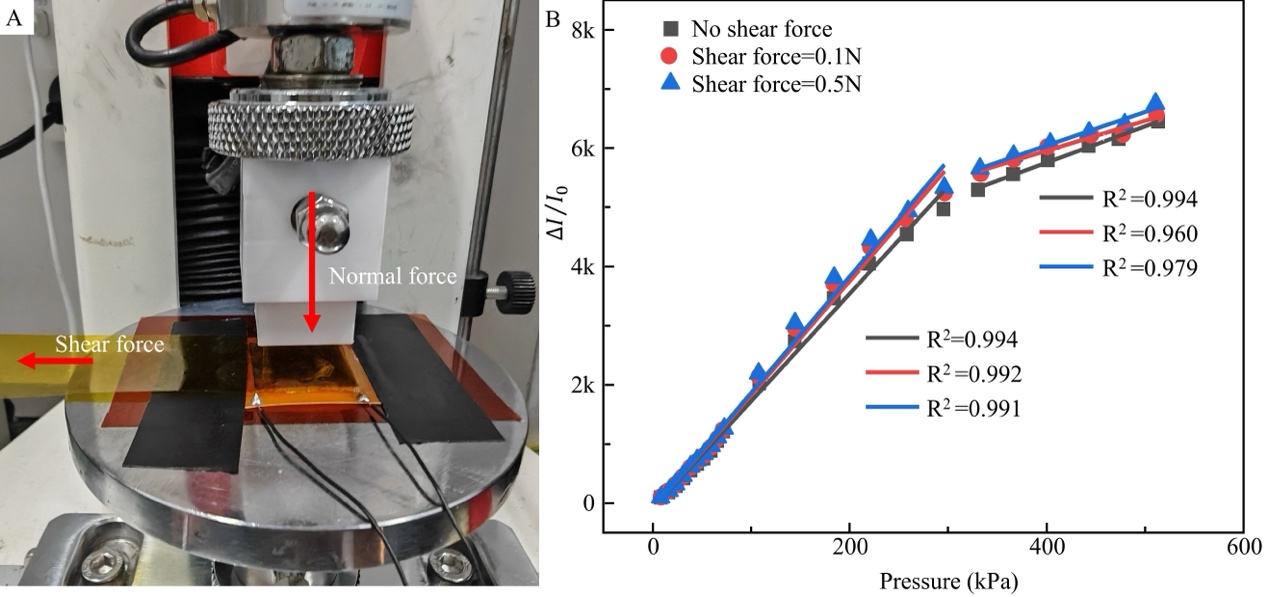


Fig. S10. Measuring setup (A) and measurement results (B) under the influence of tangential force.


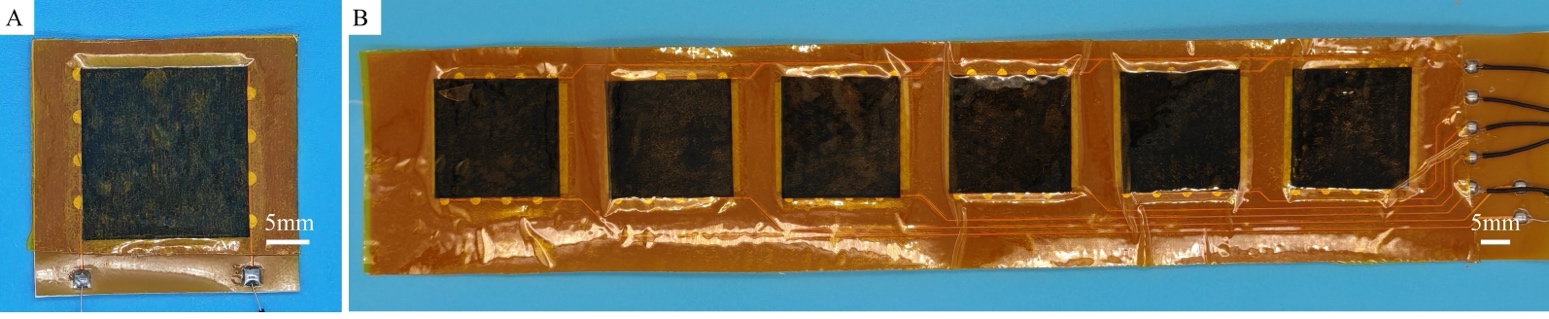


Fig. S11. Optical photos of sensor units and arrays.


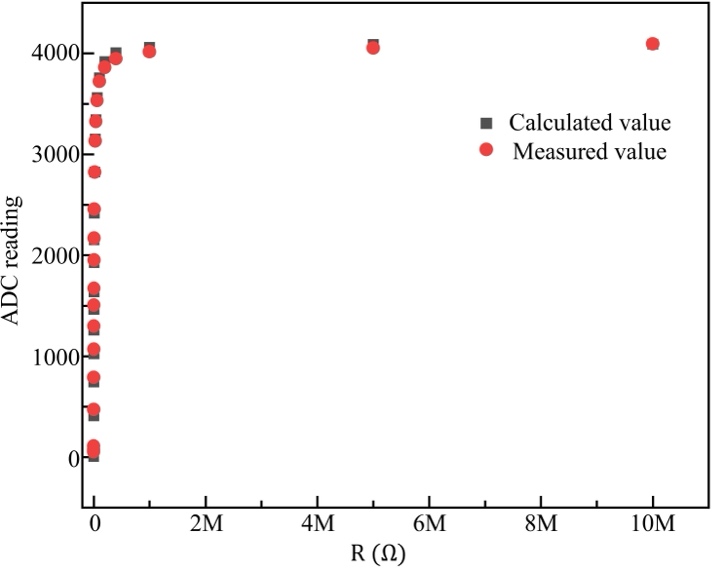


Fig. S12. Comparison between theoretical calculation and measured values of hardware acquisition system.


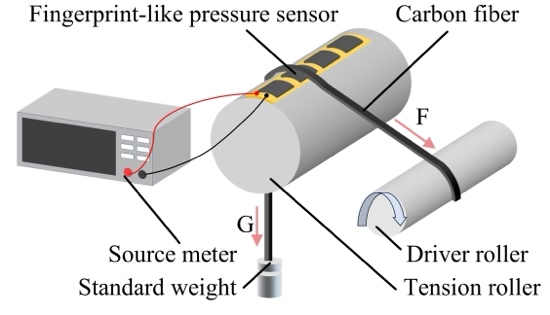


Fig. S13. Schematic diagram of the device for measuring constant standard tow tension.


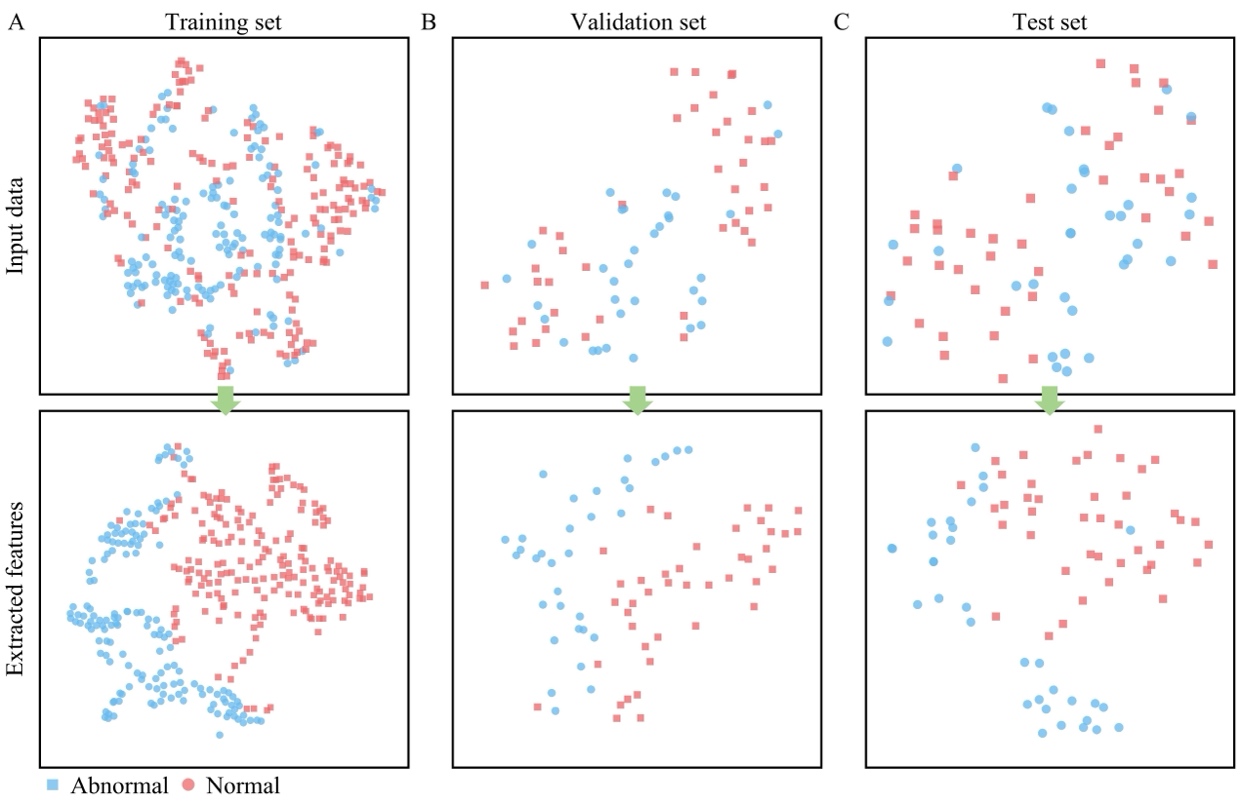


Fig. S14. The t-SNE distributions of the raw data and extracted features for the training (A), validation (B), and test sets (C), respectively.


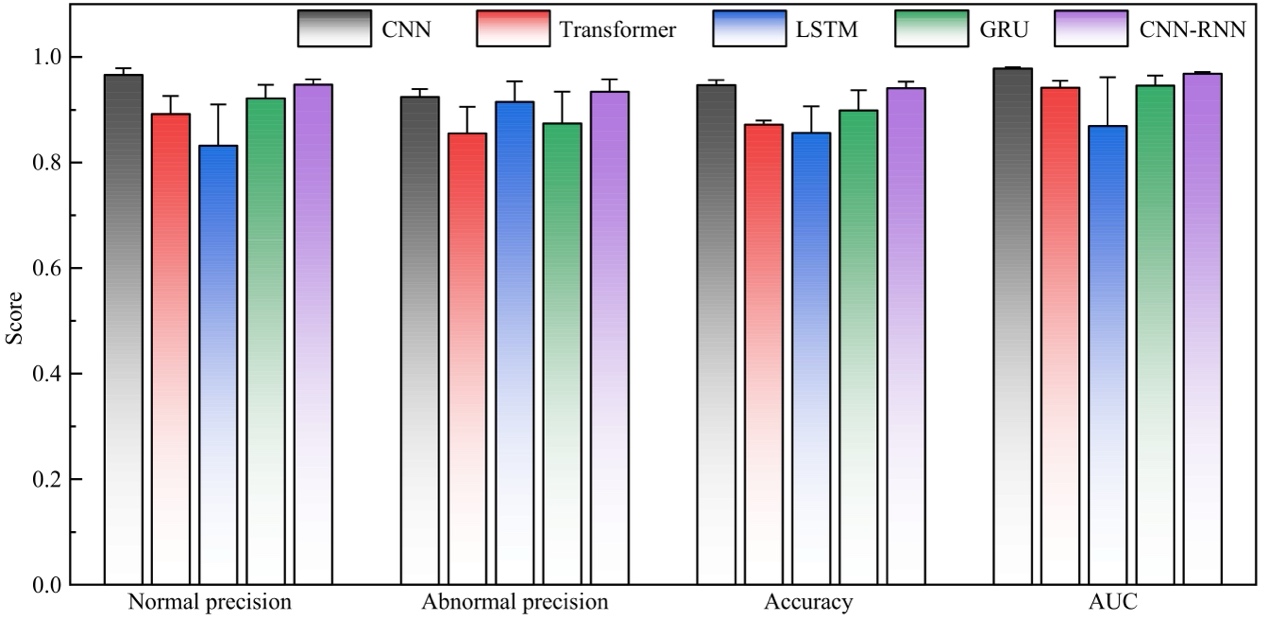


Fig. S15. Comparison of the proposed CNN with other models.

Table S1. Detailed process parameters of laser engraving fingerprint-like sensing layer.

| Parameter | Value | Unit |
| --- | --- | --- |
| Output power | 10 | W |
| Minimum light spot diameter | 50 | Μm |
| Laser beam wavelength | 1064 | Nm |
| Pulse width | 100 | Ns |
| Output bandwidth | 5 | Nm |
| Focus height | 18 | cm |
| Laser scan speed | 200 | mm/s |
| Laser spacing | 20 | μm |
| Number of passes | 3 | - |
| Repetition rate | 20 | Khz |

Table S2. Four types of interdigital structural parameters.

| Type | Electrode width *W*（mm） | Number of electrode pairs | Filling factor | Width gap ratio *W/g* |
| --- | --- | --- | --- | --- |
| A | 0.75 | 7 | 30% | 0.43 |
| B | 1 | 7 | 40% | 0.67 |
| C | 1.5 | 7 | 60% | 1.50 |
| D | 4 | 4 | 77% | 3.35 |

Table S3. Performance comparison between the developed sensor and other piezoresistive flexible pressure sensors.

| Sensitivity (kPa^-1^) | Durability | Ref. |
| --- | --- | --- |
| 10.41(0-2.5kPa), 1.8(2.5-32kPa) | 10000 | 29 |
| 36.66(0-0.6kPa), 5.11(0.6-5kPa), 2.12(5-300kPa) | 5000 | 30 |
| 11.06(0.6-10 kPa), 4.5(10-30 kPa) | 1000 | 31 |
| 15.4(0-200 kPa) | 7500 | 32 |
| 13.7(0-12kPa) | 10000 | 33 |
| 30.8(12-100kPa), 9.7(100-200kPa) | 15000 | 34 |
| 33.95(0-80kPa) | 1000 | 35 |
| 18.08(5-320kPa), 5.46(320-550kPa) | 25000 | This work |

Table S4. Detailed architecture of the developed anomaly detection model.

| Layer | Type | Output shape | **Parameter** |
| --- | --- | --- | --- |
| 1 | Conv1d (kernel size=3, padding=1, stride=1) | (B, 32, 120) | 320 |
| 2 | BatchNorm1d | (B, 32, 120) | 64 |
| 3 | Dropout (p=0.7) | (B, 32, 120) | 0 |
| 4 | ReLU | (B, 32, 120) | 0 |
| 5 | Conv1d (kernel size=3, padding=1, stride=1) | (B, 32, 120) | 3,104 |
| 6 | BatchNorm1d | (B, 32, 120) | 64 |
| 7 | Dropout (p=0.5) | (B, 32, 120) | 0 |
| 8 | ReLU | (B, 32, 120) | 0 |
| 9 | Conv1d (kernel size=3, padding=1, stride=1) | (B, 32, 120) | 3,104 |
| 10 | BatchNorm1d | (B, 32, 120) | 64 |
| 11 | Dropout (p=0.3) | (B, 32, 120) | 0 |
| 12 | ReLU | (B, 32, 120) | 0 |
| 13 | AdaptiveAvgPool1d | (B, 32, 1) | 0 |
| 14 | Linear | (B, 256) | 8,448 |
| 15 | Dropout (p=0.7) | (B, 256) | 0 |
| 16 | ReLU | (B, 256) | 0 |
| 17 | Linear | (B, 2) | 514 |
| **Total parameter** | | | **15,682** |

Table S5. Summary of the anomaly detection model performance (mean ± SD) over 5-fold cross-validation on the test set.

|  | Precision | Recall | F1-score | Support |
| --- | --- | --- | --- | --- |
| Normal | 0.9658±0.0128 | 0.9381±0.0130 | 0.9517±0.0085 | 42 |
| Abnormal | 0.9242±0.0148 | 0.9576±0.0166 | 0.9405±0.0106 | 33 |
| Macro avg | 0.9450±0.0095 | 0.9478±0.0097 | 0.9461±0.0095 | 75 |
| Weighted avg | 0.9475±0.0094 | 0.9467±0.0094 | 0.9468±0.0094 | 75 |
| Accuracy | | | 0.9467±0.0094 | 75 |
| AUC score | | | 0.9779±0.0025 |  |

Movie S1. Cyclic testing video of the sensor.
